# Measure of Innovation: From Clinical Questions to Actionable Evidence

**DOI:** 10.1002/cai2.70086

**Published:** 2026-10-14

**Authors:** Shouzheng Wang, Fei Ma

**Affiliations:** ^1^ State Key Laboratory of Molecular Oncology, National Cancer Center/National Clinical Research Center for Cancer/Cancer Hospital Chinese Academy of Medical Sciences and Peking Union Medical College Beijing China; ^2^ Department of Medical Oncology, State Key Laboratory of Molecular Oncology, Beijing Key Laboratory of Innovative Drug Research and Translational Study for Breast Cancer, National Cancer Center/National Clinical Research Center for Cancer/Cancer Hospital Chinese Academy of Medical Sciences and Peking Union Medical College Beijing China

**Abbreviation**
IITs: investigator‐initiated trials

1

Anticancer drug development has entered an age of abundance, but also one of unfinished promises. New targets, mechanisms, therapeutic agents, and treatment modalities are emerging at a remarkable pace. However, their scientific promise does not always translate into durable clinical benefits. Many patients still experience inadequate responses, treatment‐related toxicities, or limited treatment options after disease progression, while clinically important patient subgroups remain underserved by current care. The clinical relevance of a development program therefore does not begin with how novel an intervention appears, but with the unmet need it seeks to address in a defined patient population.

Novelty has its allure. A first‐in‐class agent may open a field or reveal previously inaccessible vulnerabilities. However, scientific distinction and clinical value are not the same achievement. First‐in‐class status marks innovation but does not, by itself, demonstrate added benefit for patients [1]. Drug development begins to matter when scientific possibility is directed toward a clinical problem worth solving.

## 2 The Question Worth Asking

A meaningful development program begins with a question that matters to patients. Why does an effective therapy fail too soon? Why does a particular subgroup derive little benefit? Can efficacy be preserved with less toxicity or recovered once resistance appears? Such questions give direction to discovery and determine what evidence will ultimately matter.

The challenge lies in turning a clinical problem into a testable therapeutic hypothesis. A detectable molecular alteration may suggest a target, but does not make that target clinically actionable. A mechanistically appealing combination may justify investigation, but does not establish synergy. A new modality may broaden what is technically possible without yet indicating whom it should treat or whether it improves the balance of benefit and harm.

Therefore, a more useful discipline would be to ask what uncertainty must be reduced before a clinical decision can reasonably change. That uncertainty may involve patient selection, mechanism, resistance, dose, treatment sequence, or combination strategy. The question should come first. Technology and novelty should serve it.

## 3 Evidence Worth Acting On

Early clinical development is where biological possibility first faces clinical scrutiny. Mechanism, exposure, pharmacodynamic effects, safety, tolerability, and preliminary antitumor activity should be considered concurrently rather than as isolated signals.

The phase I development of pyrotinib in human epidermal growth factor receptor 2 ‐positive metastatic breast cancer illustrates this approach. Dose escalation, pharmacokinetics, safety, antitumor activity, and predictive biomarkers were all evaluated within the same clinical framework, with next‐generation sequencing performed on circulating tumor DNA and tumor tissue [2]. The study established a maximum tolerated dose while also providing early evidence on drug exposure, clinical activity, and molecular correlates of response. Its value does not lie in any single endpoint but in how these signals begin to form an argument for what should happen next.

Early signals, however, are easily overread. Target engagement does not necessarily predict tumor control, nor does an early response necessarily translate into longer survival or improved quality of life. An endpoint is informative only in relation to the clinical setting and the question a study is designed to address [3]. The pharmacological audit trail provides a similar perspective, viewing patient selection, pharmacokinetics, pharmacodynamics, response, and resistance as interdependent elements of drug development [4].

This integrated view becomes particularly important when deciding whom to treat. Biomarkers identified in early studies are usually provisional and gain clinical meaning only when supported by tumor biology and observed treatment response. Whenever feasible, molecular assessment should extend beyond baseline, because responses and resistance may alter how the original biomarker should be interpreted.

Dose selection raises another question. The maximum tolerated dose is not always an adequate guide for agents whose efficacy and toxicity do not rise in parallel. Dose and schedule are themselves clinical hypotheses. A randomized phase II study comparing the efficacy, safety, and pharmacokinetics of 960 and 240 mg of sotorasib for KRAS G12C‐mutated advanced non‐small cell lung cancer illustrates how dose selection can itself become a prospective clinical question [5]. The dose worth advancing should therefore be supported by evidence on both activity and tolerability, rather than assumed from the highest dose that can be administered.

Evidence becomes meaningful when it changes the next decision. It may support further development, narrow the population, revise the dose, redirect the study, or bring a program to an end. Negative findings belong to this process. Knowing when to change course is as important as knowing when to move forward.

## 4 Questions Emerging From Evidence

Clinical investigation does not travel in one direction from laboratory discovery to bedside application. What is learned in patients often returns as the next scientific question. An exceptional response may uncover an unrecognized dependency. Primary resistance can expose weaknesses in the original biological model, whereas acquired resistance may reveal vulnerabilities that were not apparent at baseline. Toxicity can likewise prompt reconsideration of dose, schedule, or combination strategies.

Linking clinical outcomes with pretreatment, on‐treatment, and progression samples can translate these observations into the next testable question. The bedside can therefore become the starting point for discoveries and the setting in which they are tested.

This is where clinically driven investigator‐initiated trials (IITs) can be particularly informative. Important questions often emerge only after a therapy reaches clinical testing or routine practice, whether they concern a biomarker‐defined subgroup, resistance, rational combinations, treatment sequencing, or dose and schedule. Addressing these questions may require no new drugs at all but rather a rigorous prospective study built around a clinically meaningful hypothesis.

A relevant example is an investigator‐led randomized phase II trial in human epidermal growth factor receptor two‐negative metastatic breast cancer. Bayesian adaptive randomization was used to evaluate metronomic and conventional chemotherapy strategies, including programmed cell death protein 1 blockade‐containing regimens, while mass cytometry examined associated immune changes [6]. The significance of this study extends beyond the regimen itself. It demonstrates how a clinically derived question, an adaptive design, and translational investigation can be integrated within the same prospective trial.

IITs are not inherently superior to industry‐sponsored studies. Their strength lies in the questions they can pursue. When an important uncertainty falls outside the conventional route of drug development, a well‐designed prospective IIT can return that question to systematic testing.

For the same reason, phase labels should not become rigid boundaries around types of evidence. What matters is not simply where a study is positioned within the conventional sequence of development but what uncertainty it reduces and what decision its evidence can support.

## 5 A Forum for Questions Worth Pursuing

This Special Issue brings together these related forms of evidence generation. We welcome early‐phase studies on innovative anticancer agents and clinically driven prospective IITs, including work on new targets and mechanisms, emerging modalities, patient selection, resistance, rational combinations, treatment sequencing, and dose optimization.

Anticancer innovation fulfills its promise when biological possibility becomes a meaningful clinical choice. The question worth asking is one rooted in unmet need. The evidence worth acting on is evidence strong enough to change what comes next. Meaningful evidence does not end inquiry. It sharpens the question that follows.

Innovation begins with what science makes possible. Progress depends on knowing what is worth pursuing, what evidence is sufficient to act, and when what we have learned asks us to look again.

## Author Contributions

**Shouzheng Wang:** writing – original draft (lead), conceptualization (supporting). **Fei Ma:** conceptualization (lead), writing – review and editing (lead), funding acquisition (lead), supervision (lead).

## Ethics Statement

The authors have nothing to report.

## Consent

The authors have nothing to report.

## Conflicts of Interest

Professor Fei Ma is a member of the *Cancer Innovation* Editorial Board. To minimize bias, he was excluded from all editorial decision‐making related to the acceptance of this article for publication. The remaining author declares no conflicts of interest.

## Data Availability

The authors have nothing to report.
